# 6-Azauridine Induces Autophagy-Mediated Cell Death via a p53- and AMPK-Dependent Pathway

**DOI:** 10.3390/ijms22062947

**Published:** 2021-03-14

**Authors:** Yeo-Eun Cha, Rackhyun Park, Minsu Jang, Yea-In Park, Ayane Yamamoto, Won Keun Oh, Eun-Ju Lee, Junsoo Park

**Affiliations:** 1 Division of Biological Science and Technology, Yonsei University, Wonju 26493, Korea; ckdudms@yonsei.ac.kr (Y.-E.C.); rockhyun@yonsei.ac.kr (R.P.); minsujang@yonsei.ac.kr (M.J.); pyi012324@yonsei.ac.kr (Y.-I.P.); ayane.yamamoto@yonsei.ac.kr (A.Y.); 2 Korea Bioactive Natural Material Bank, Research Institute of Pharmaceutical Sciences, College of Pharmacy, Seoul National University, Seoul 08826, Korea; wkoh1@snu.ac.kr; 3 Department of Obstetrics and Gynecology, Chung-Ang University School of Medicine, Seoul 06974, Korea; ejlee@cau.ac.kr

**Keywords:** autophagy-mediated cell death, 6-azauridine, autophagy, autophagic flux

## Abstract

6-Azauridine (6-AZA), a pyrimidine nucleoside analogue, is known to exhibit both antitumor and antiviral activities. Although 6-AZA was discovered more than 60 years ago, the cellular effects of this compound are yet to be elucidated. Here, we report that 6-AZA regulates autophagy-mediated cell death in various human cancer cells, where 6-AZA treatment activates autophagic flux through the activation of lysosomal function. Furthermore, 6-AZA exhibited cytotoxicity in all cancer cells studied, although the mechanisms of action were diverse. In H460 cells, 6-AZA treatment induced apoptosis, and the extent of the latter could be reduced by treatment with chloroquine (CQ), a lysosomal inhibitor. However, 6-AZA treatment resulted in cell cycle arrest in H1299 cells, which could not be reversed by CQ. The cytotoxicity associated with 6-AZA treatment could be linearly correlated to the degree of autophagy-mediated cell death. In addition, we demonstrated that the cytotoxic effect of 6-AZA was dependent on AMPK and p53. These results collectively indicate that autophagy-mediated cell death triggered by 6-AZA contributes to its antitumor effect.

## 1. Introduction

Autophagy, originally identified as a protein degradation pathway, is closely involved in cell death; however, the role of autophagy in cell death remains controversial [1]. The relationship between autophagy and cell death is dependent on cellular context, and both excessive and reduced levels of autophagy contribute to cell death [2,3]. Cell death with excessive autophagosome formation is commonly referred to as autophagic cell death; however, excessive formation of autophagosomes is not always associated with cell death [4]. Numerous reports have demonstrated that autophagosomes can confer protection to cells from stress-induced cell death [5,6]. In addition, treatment with chloroquine (CQ), an autophagic flux inhibitor, often leads to increased cell death by suppressing the protective role of autophagy in cellular physiology [7].

When autophagy activates cell death, its role can be classified as autophagy-associated cell death, autophagy-mediated cell death, and autophagy-dependent cell death [8]. Autophagy-associated cell death is characterized by autophagy induction, along with apoptosis activation, where autophagy is not involved in cell death. Autophagy-mediated cell death is the induction of cell death, including apoptosis, which is dependent on autophagy activation [8]. Finally, autophagy-dependent cell death is defined as an autophagy-driven cell death process that does not require apoptosis and necrosis induction [9]. Autophagy-mediated cell death is frequently induced by stress, such as drug treatment [10,11].

6-Azauridine (6-AZA) was originally identified as a metabolite of 6-azauracil in bacteria, and is now known to exhibit antitumor activity against human tumors, such as leukemia, as well as in animal tumor models [12,13]. A previous study reported that the antitumor activity of 6-AZA is mediated by the inhibition of the pyrimidine de novo synthesis pathway, with the addition of pyrimidine nucleotides observed to recover 6-AZA-induced cytotoxicity [12,14]. However, 6-AZA was not effective in the treatment of human tumors, possibly due to the transient nature of its action. 6-AZA is preferentially incorporated into the RNA of non-mammalian cells, such as viruses and bacteria, thereby exhibiting antiviral and antibacterial activities. 6-AZA is active against influenza viruses, flaviviruses, and coronaviruses [15,16,17].

Here, we report that 6-AZA treatment induces autophagy and reduces cell viability. Since CQ blocks autophagic flux, we assumed that CQ-induced reduction in cytotoxicity represents autophagy-mediated cell death. We further found that differential levels of autophagy-mediated cell death and 6-AZA cytotoxicity in various cancer cell lines are partly dependent on the presence of p53 and AMPK.

## 2. Results

### 2.1. 6-AZA Activates Autophagic Flux in Cancer Cells

**Figure 1:**
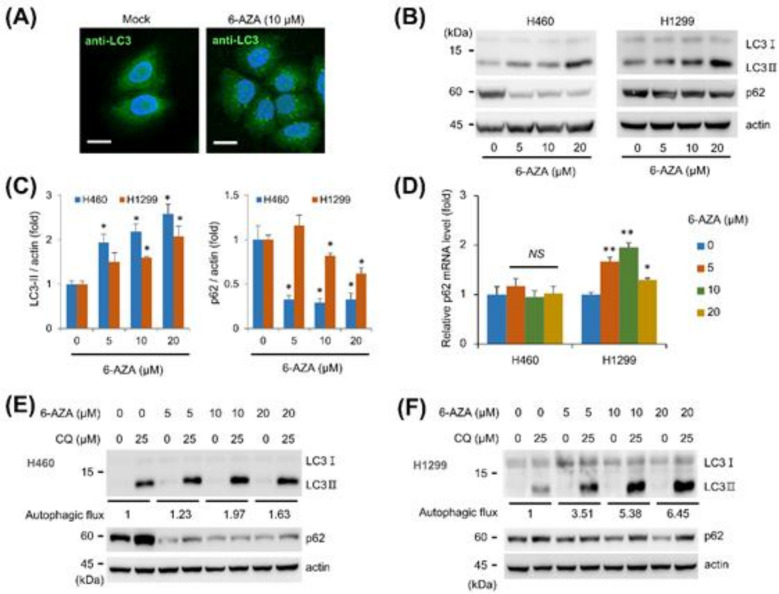
6-Azauridine (6-AZA) activates autophagic flux. (**A**) 6-AZA treatment induces autophagosome formation. H460 cells were treated with 6-AZA (10 μM) for 24 h and immunostained with anti-LC3 antibody. Bars, 20 μm. (**B**) 6-AZA treatment results in elevated LC3-II levels. H460 and H1299 cells were treated with the indicated concentration of 6-AZA for 24 h, and cell lysates were subjected to Western blotting with the indicated antibodies. (**C**) Expression levels of LC3-II and p62 were analyzed in H460 and H1299 cells. Experiments were performed at least in triplicate, and values of the mean and standard error are shown in the graph. Mock vs. drug treatment. * *p* < 0.05. (**D**) p62 mRNA level was evaluated by qRT-PCR. Mock vs. drug treatment. * *p* < 0.05, ** *p* < 0.005, *NS* not significant. (**E**) 6-AZA treatment induces autophagic flux in H460 cells. Cells were treated with the indicated concentration of 6-AZA in the presence or absence of chloroquine (CQ) for 24 h, and cell lysates were subjected to Western blotting with the indicated antibodies. Autophagic flux was calculated from the difference between control and CQ treatments. (**F**) 6-AZA treatment induced autophagic flux in H1299 cells.

We examined several molecules for their autophagy-regulating activity and identified the ability of 6-AZA to induce autophagosomes (data not shown). We checked for autophagosome formation in lung cancer cell lines H460 upon 6-AZA treatment by staining the cells with an anti-LC3 antibody (Figure 1A). Next, we confirmed that 6-AZA regulated the autophagic process by analyzing LC3-II and p62 protein expression levels. 6-AZA treatment increased the level of LC3-II protein, indicating that 6-AZA induces autophagosome formation (Figure 1B,C). While the level of p62 protein in H460 cells was drastically decreased upon 6-AZA treatment, the decrease was more subtle in H1299 cells, suggesting that the effect of 6-AZA on p62 expression is different in different cancer cells (Figure 1B,C). Since p62 expression patterns were different in H460 and H1299 cells, we next examined the mRNA expression of p62 upon 6-AZA treatment. Quantitative RT-PCR (qRT-PCR) showed modest changes in p62 mRNA levels in H460 cells, and an induction of p62 mRNA expression in H1299 cells (Figure 1D). We next examined autophagic flux by blocking lysosomal function; CQ treatment was found to increase LC3-II expression in the presence of 6-AZA in H460 and H1299 cells, indicating that autophagic flux is activated by 6-AZA treatment (Figure 1E,F). These results collectively indicate that 6-AZA treatment induces autophagosome formation and activates autophagic flux.

### 2.2. 6-AZA Activates Lysosomal Function in Cancer Cells

**Figure 2:**
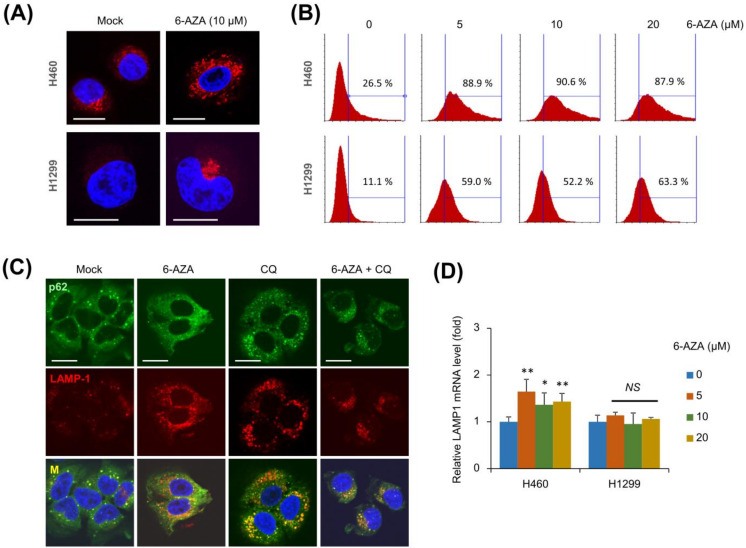
6-AZA treatment activates lysosomal function. (**A**) 6-AZA treatment led to enhanced LysoTracker staining in H460 and H1299 cells. 6-AZA-treated cells were stained with LysoTracker and DAPI and imaged by confocal microscopy. Bars, 20 μm. (**B**) 6-AZA-treated cells were stained with LysoTracker and analyzed by flow cytometry. (**C**) H460 cells were treated with 6-AZA (10 μM) in combination with CQ (25 μM), and immunostained with anti-p62 antibody and anti-LAMP1 antibody. Bars, 20 μm. (**D**) *LAMP1* mRNA level in 6-AZA-treated cells was analyzed by qRT-PCR. Mock vs. drug treatment. * *p* < 0.05, ** *p* < 0.005, *NS* not significant.

Since 6-AZA treatment induces autophagic flux, we examined whether lysosomal function is activated by 6-AZA treatment. We treated H460 cells with 6-AZA and stained the cells with LysoTracker dye, which stains the acidic lysosome [18]. Microscopy-based analysis showed that 6-AZA treatment led to enhanced LysoTracker staining in H460 and H1299 cells (Figure 2A). Moreover, flow cytometry analysis indicated that the number of cells with acidic lysosomes increased (Figure 2B). Next, we immunostained p62 and LAMP1 proteins in H460 cells treated with 6-AZA and CQ. 6-AZA increased the expression of LAMP-1, similar to that observed in CQ treatment (Figure 2C). *LAMP-1* mRNA expression was also significantly increased in 6-AZA treatment in H460 cells, whereas no changes in *LAMP-1* mRNA were observed in H1299 cells (Figure 2D). These results indicate that 6-AZA activates lysosomal function.

### 2.3. 6-AZA Treatment Induces Autophagy-Mediated Cell Death

**Figure 3:**
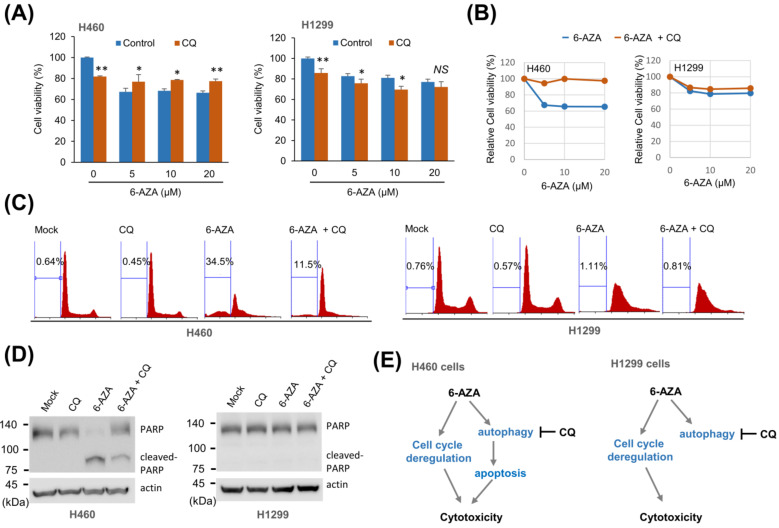
6-AZA exerts antitumor activity by regulating the autophagy–apoptosis link. (**A**) 6-AZA treatment decreased cell viability of H460 and H1299 cells treated with indicated concentrations of 6-AZA in the presence or absence of CQ. Cell viability was measured using an MTT assay. 6-AZA alone vs. combined treatment with 6-AZA and CQ. * *p* < 0.05, ** *p* < 0.005, *NS* not significant. (**B**) Relative cell viability was plotted by setting MTT activity without drug treatment as 100%. (**C**) 6-AZA treatment induced apoptosis and/or cell cycle dysregulation in H460 and H1299 cells treated with 6-AZA, in combination with CQ, for 24 h. Cell cycle progression was analyzed by flow cytometry. H460 cells showed an increased sub-G1 population. (**D**) H460 and H1299 cells were treated with 6-AZA in combination with CQ for 24 h, and the extent of PARP cleavage was examined. H460 cells showed cleaved PARP upon 6-AZA treatment, whereas H1299 cells did not. (**E**) Schematic diagram showing the distinctive cytotoxic mechanism of 6-AZA in H460 and H1299 cells.

Since 6-AZA has been reported to act as an antitumor drug, we examined whether 6-AZA induces cell death. 6-AZA treatment decreased cell viability in H460 and H1299 cells, with the H460 cell line exhibiting greater sensitivity to 6-AZA treatment (Figure 3A). Interestingly, when cells were treated with 6-AZA in the presence of CQ, the effect was different. 6-AZA treatment did not decrease viability of H460 cells in the presence of CQ; however, 6-AZA treatment in the presence of CQ decreased cell viability, similar to 6-AZA alone (Figure 3B). Next, we checked for changes in cell cycle progression following 6-AZA treatment. 6-AZA treatment in H460 cells resulted in an increased sub-G1 population, indicating apoptosis induction, and both 6-AZA and CQ treatment resulted in decreased levels of sub-G1 population, indicating that the overall level of apoptosis had decreased (Figure 3C). On the other hand, 6-AZA treatment did not induce an increase in sub-G1 population in H1299 cells; although, an increased proportion of cells was observed in the S phase, suggesting an inhibitory effect of 6-AZA treatment on cell cycle progression (Figure 3C). In addition, CQ treatment could not reverse cell cycle inhibition, similar to that observed in the control (Figure 3C). Next, we confirmed the induction of apoptosis by examining PARP cleavage in these cells. While 6-AZA treatment induced PARP cleavage, indicating apoptosis induction, combined treatment with 6-AZA and CQ reduced the extent of PARP cleavage (Figure 3D). However, we did not detect any cleaved form of PARP protein in H1299 cells (Figure 3D). These results indicate that the inhibitory effect of 6-AZA on cancer cells is diverse and cell-type dependent (Figure 3E).

### 2.4. 6-AZA Shows Diverse Cytotoxicity to Various Cancer Cells

**Figure 4:**
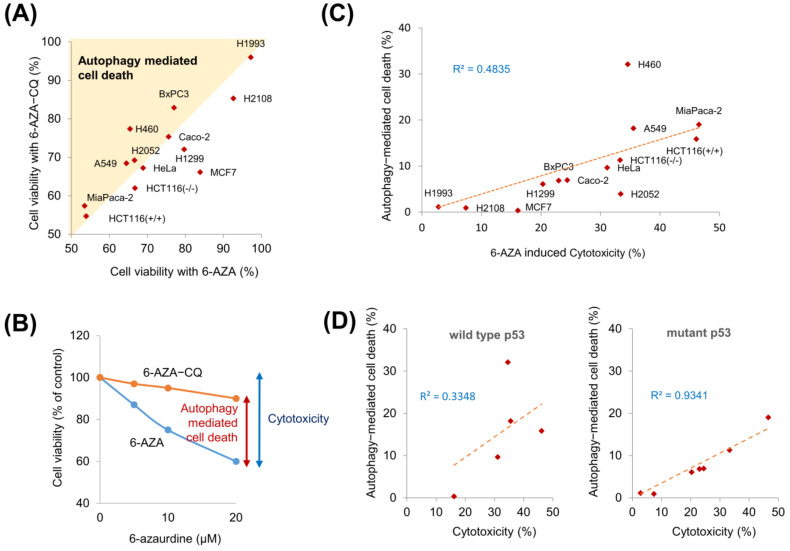
Autophagy-mediated cell death contributes to 6-AZA-induced cytotoxicity. (**A**) CQ treatment was found to modulate viability of cancer cells. Various cancer cells were treated with 6-AZA in the presence or absence of CQ, and cell viability was estimated. Cell viability on combined 6-AZA and CQ treatment was higher than that with 6-AZA alone, indicating that 6-AZA induces autophagy-mediated cell death. (**B**) Schematic representation of autophagy-mediated cell death. (**C**) Autophagy-mediated cell death and cytotoxicity were plotted, and the correlation coefficient was calculated. (**D**) Relationship between autophagy-mediated cell death and cytotoxicity was analyzed based on the p53 status (wild type vs. mutant).

Since 6-AZA treatment showed differential cytotoxicity in H460 and H1299 cells, we further examined its cytotoxicity in various other cancer cell lines. We treated cells with only 6-AZA and with a combination of 6-AZA and CQ to examine cell viability (Figure S1). We found that CQ treatment reduced 6-AZA-induced cytotoxicity in several cancer cell lines (Figure 4A). Here, we define the difference between the cytotoxicity of 6-AZA alone and the cytotoxicity of the 6-AZA and CQ combination as “autophagy-mediated cell death”, since CQ treatment blocks autophagic flux (Figure 4B). The extent of autophagy-mediated cell death was observed to be of a diverse range in the various cancer cells investigated here. We found that 6-AZA-mediated cytotoxicity tended to be higher when autophagy-mediated cell death was higher (Figure 4C). To visualize this tendency, we replotted the acquired data as a correlation graph, with the X- and Y-axes representing cytotoxicity and autophagy-mediated cell death, respectively. The correlation coefficient (R^2^) was calculated to be 0.4835, indicating that these two parameters are moderately correlated (Figure 4C). Since p53 mutation status is known to be crucial in determining the pathophysiology of cancer cells, we further sorted the data based on p53 mutation. Surprisingly, the R^2^ value obtained was appreciably high (0.9341) in p53-mutant cells and relatively low (0.3348) in p53 wild-type cells (Figure 4D). These results indicate that p53 is involved in 6-AZA-induced cell death.

### 2.5. p53 and AMPK Affect Cellular Response to 6-AZA Treatment

**Figure 5:**
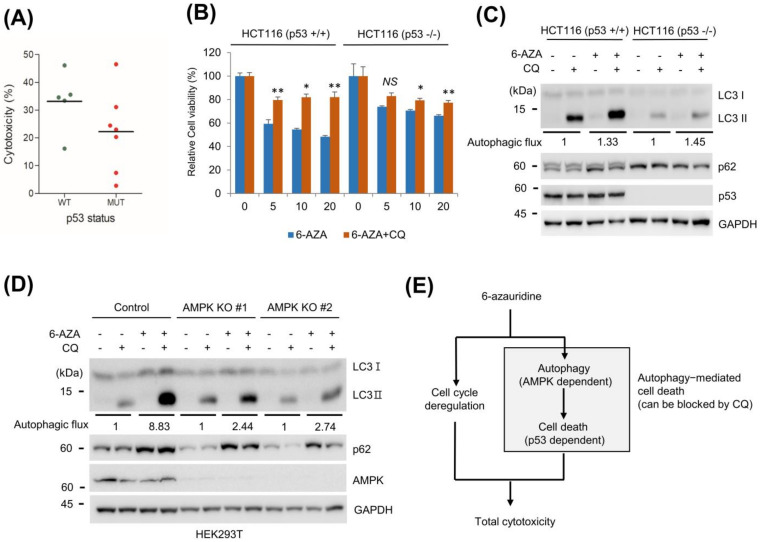
Contributions of p53 and AMPK to 6-AZA-induced autophagic flux and cell viability. (**A**) Cell viability of various cancer cells upon 6-AZA (20 μM) treatment was sorted based on p53 status (wild type vs. mutant). (**B**) p53 wild-type HCT116 (HCT116 p53 +/+) and p53 null HCT116 cells (HCT116 p53 −/−) cells were treated with the indicated concentration of 6-AZA for 24 h, and relative cell viability was measured by an MTT assay. Control vs. CQ treatment, * *p* < 0.05, ** *p* < 0.005. (**C**) HCT116 p53 +/+ and HCT116 p53 −/− cells were treated with the indicated concentration of 6-AZA (10 μM) in the presence or absence of CQ (25 μM) for 24 h, and cell lysates were subjected to Western blotting with the indicated antibodies. (**D**) AMPK knockout was found to contribute to autophagic flux induced by 6-AZA treatment. Control and AMPK KO cells were treated with 6-AZA (10 μM) in combination with CQ (25 μM), and cell lysates were subjected to Western blotting with the indicated antibodies. (**E**) 6-AZA induces autophagy-mediated cell death in an AMPK- and p53-dependent manner.

When we measured cell viability upon 6-AZA treatment based on the p53 mutation status, median cell viability of cancer cells containing wild-type p53 was found to be lower than that of p53 mutant cancer cells (Figure 5A). Therefore, we next examined the effect of p53 on cell viability upon 6-AZA treatment. We used HCT116 cells containing p53 wild-type (HCT116 p53 +/+) and p53 null HCT116 cells (HCT116 p53 −/−). We found that HCT116 p53 +/+ cells were more sensitive to 6-AZA treatment than HCT116 p53 −/− cells (Figure 5B). In addition, the difference in viability associated with only 6-AZA treatment and combined 6-AZA and CQ treatment was higher (Figure 5B). These results indicate that p53 contributes to changes in cell viability observed upon 6-AZA treatment. Next, we examined 6-AZA-induced differences in autophagic flux based on p53 mutation status. Basal autophagic flux was higher in wild-type p53 HCT116 cells compared to that in p53 null HCT116 cells; however, the increase in basal flux in 6-AZA treatment was not distinctive (Figure 5C). These results indicate that the presence of p53 contributes largely to 6-AZA-induced cytotoxicity.

AMPK is reported to be crucial for autophagy initiation and autophagic flux [19]. Control wild-type and AMPK knockout (KO) HEK 293T cells were used to evaluate the effect of AMPK on 6-AZA-induced autophagic flux. The autophagic flux in control cells on 6-AZA treatment was stronger than that in AMPK KO cells in the presence of CQ (Figure 5D). In addition, AMPK KO cells were found to be more resistant to 6-AZA treatment (Figure S2). These results collectively indicate that AMPK is involved in 6-AZA-induced autophagic flux, and hint at the involvement of p53 in cell death (Figure 5E).

## 3. Discussion

In this study, we demonstrate that 6-AZA induces autophagy and decreases cell viability in cancer cells. Since 6-AZA is reported to have antitumor activity, we assumed that the regulation of autophagy by 6-AZA contributes to antitumor actions. We examined various cancer cells and found that the effect of 6-AZA is diverse in different cancer cells. This differential effect is caused by the fact that the genetic background of each cancer cell line studied was different. Here, we attempted to identify the common elements that contribute to the differential extent of autophagy and cell viability in 6-AZA treatment of different cancer cells.

6-AZA treatment was observed to induce autophagosome formation and activate autophagic flux, in addition to activating lysosomal function. Lysosomal digestive enzymes are known to be activated in acidic lysosomal conditions, and the LysoTracker dye was used to stain these acidic lysosomes. Both microscopy-based analysis and flow cytometry assays showed that 6-AZA leads to increased lysosomal staining, indicating the activation of lysosomal function by 6-AZA (Figure 2A,B). Next, we examined the expression of *LAMP1* mRNA upon 6-AZA treatment. While 6-AZA treatment induced *LAMP1* mRNA expression in H460 cells, this effect was observed only to a moderate extent in H1299 cells (Figure 2D). Changes in both lysosomal staining and *LAMP1* mRNA expression levels suggest that H460 cells have more active lysosomes.

6-AZA treatment exerted cytotoxic effects by inducing apoptosis or dysregulating the cell cycle. Since cancer cells are characterized by diverse genetic backgrounds, the effect of 6-AZA on cell proliferation was found to depend on the specific cancer cell line studied. 6-AZA was found to induce apoptosis in H460 cells by increasing the sub-G1 population and the cleaved form of PARP. CQ treatment, intended to suppress the 6-AZA-induced autophagic flux, led to a decreased level of apoptosis. These results indicate that autophagy contributes to 6-AZA-induced apoptosis (Figure 3). On the other hand, the sub-G1 population or cleaved form of PARP was not increased in H1299 cells. Instead, we observed a dramatic increase in the number of S phase cells in the H1299 cell line upon 6-AZA treatment, indicating that 6-AZA dysregulates the cell cycle. Moreover, this effect could not be rescued by CQ treatment. These results indicate that autophagy induction by 6-AZA does not contribute to the cell cycle dysregulation observed in H1299 cells. We assumed that the link between autophagy and apoptosis, in terms of cellular signaling cascades, is different in H460 and H1299 cells (Figure 3E). In other words, while CQ treatment increased the viability of H460 cells by blocking the autophagy–apoptosis link, CQ treatment did not increase the viability of H1299 cells, suggesting that the autophagy–apoptosis link is missing in H1299 cells (Figure 3E).

Since apoptosis and cell cycle dysregulation contribute to cellular cytotoxicity, we hypothesized that 6-AZA would be more toxic to cancer cells where both apoptosis induction and cell cycle dysregulation are triggered by 6-AZA. To prove our hypothesis, we calculated the extent of autophagy-mediated cell death by measuring the difference in cytotoxicity induced by 6-AZA only and the combination of 6-AZA and CQ; we found a correlation between cytotoxicity and autophagy-mediated cell death (Figure 4B,C). When autophagy-mediated cell death was higher, cytotoxicity was also higher, suggesting that autophagy-mediated cell death contributes to cytotoxicity (Figure 4C).

On testing the viability of various cancer cells upon 6-AZA treatment, cancer cells with wild-type p53 appeared to be more sensitive to 6-AZA treatment than cancer cells with mutant p53 (Figure 5A). Moreover, H460 cells expressing wild-type p53 were more sensitive to 6-AZA than H1299 cells lacking p53 expression. To confirm our hypothesis, we used HCT116 p53 +/+ and HCT116 p53 −/− cells. The cell viability assay showed that HCT116 p53 +/+ cells were more sensitive to 6-AZA treatment, indicating that the p53 mutation status contributes to 6-AZA-induced cytotoxicity.

Since AMPK is important for autophagy initiation and autophagic flux, we next examined the effect of AMPK expression on 6-AZA-induced autophagy. We used HEK293T AMPK KO cells for this purpose, and found that the autophagic flux induced in AMPK KO cells was weaker than that in control HEK293T cells. These results indicate that AMPK is important for 6-AZA-induced autophagic flux (Figure 5D).

Our observations collectively indicate that 6-AZA induces cytotoxicity by activating autophagy-mediated cell death and triggering cell cycle dysregulation (Figure 5E). 6-AZA-induced autophagy is regulated in an AMPK-dependent manner, and autophagy-mediated cell death occurs in a p53-dependent manner. Therefore, 6-AZA will be more effective for treatment of cancer cells with wild-type p53 and wild-type AMPK expressions. We speculate that other factors related to p53 and AMPK pathways may additionally contribute to 6-AZA-induced cancer cell death.

## 4. Materials and Methods

### 4.1. Cell Culture and Cell Proliferation Assay

H460, H1299, MCF-7, HCT116, MIA PaCa-2, H2108, H2199, A549, HeLa, Caco-2, and BxPC-3 cells were cultured in DMEM (Welgene, Korea) supplemented with 10% fetal bovine serum (Gibco, Grand Island, NY, USA). H2052 cells were grown in RPMI medium (Welgene) with 10% fetal bovine serum. Cell proliferation was measured using the 3-(4,5-dimethylthiazol-2-yl)-2,5-diphenyltetrazolium bromide (MTT) assay. Briefly, cells were seeded in a 24-well plate, incubated overnight, and treated with the antitumor drugs. At the indicated time, MTT solution was added to a final concentration of 1 mg/mL, and the mixture was incubated for an additional 3 h. MTT was purchased from USB Corporation (Cleveland, OH, USA). 6-AZA, bafilomycin A1, and CQ were purchased from Sigma Aldrich (St. Louis, MO, USA). Natural product library was obtained from the Korea Bioactive Natural Material Bank (KBNMB).

### 4.2. Western Blot

For Western blot analysis, polypeptides in whole cell lysates were resolved by SDS-PAGE and transferred to nitrocellulose membrane filters. Detection was performed with a 1:5000 or 1:10,000 dilution of primary antibody using an enhanced chemiluminescence (ECL) system. Images were acquired using the LAS4100 system (GE Healthcare, Uppsala, Sweden). The antibodies for p62 and LC3 were purchased from MBL (Woburn, MA, USA), antibody for PARP from GeneTex (San Antonio, TX, USA), that for actin from Applied Biological Materials (Richmond, Canada), and the antibody for GAPDH from Santa Cruz Biotechnology (Santa Cruz, CA, USA).

### 4.3. Immunofluorescence Staining and Confocal Microscopy

Cells were grown on sterilized glass coverslips and fixed with 4% paraformaldehyde after treatment with 6-AZA. For immunostaining, cells were blocked with 10% goat serum in phosphate-buffered saline (PBS), stained with a 1:500 dilution of primary antibody in PBS, and then reacted with a 1:1000 dilution of Alexa 488- or Alexa 568-conjugated secondary antibody (Invitrogen, Carlsbad, CA, USA). Finally, the slides were washed three times with PBS, stained with DAPI, and mounted on glass slides using a mounting medium (Vector, Burlingame, CA, USA). Images were acquired with a Zeiss LSM 710 confocal microscope (Carl Zeiss, Oberkochen, Germany). LysoTracker was purchased from Invitrogen, and the antibody for LAMP1 was purchased from Santa Cruz Biotechnology.

### 4.4. Flow Cytometry for Cell Cycle and Lysosome Analysis

For cell cycle analysis, cells were washed with PBS and fixed with 70% ethanol. After centrifugation, cells were washed and resuspended in PBS containing 50 μg/mL propidium iodide (PI) and 10 mg/mL RNase A. FA-treated cells were analyzed using a FACSCalibur flow cytometer (Becton Dickinson Bioscience, Mountain View, CA, USA).

### 4.5. Quantitative Reverse Transcription-PCR

For quantitative RT-PCR, cells were harvested and RNA was extracted using Trizol (Thermo Fisher Scientific, Waltham, MA, USA), in accordance with the manufacturer′s instructions, and then subjected to RT-PCR using the StepOnePlus Real-Time PCR System (Thermo Fisher Scientific). The following primers were used for amplification: p62 forward 5′-ATCGGA GATCCGAGTGT-3′, p62 reverse 5′-TGGCTGTGAGCTGCTCTT-3′, LAMP1 forward 5′-AGTGGCCCTAAGAACA TGACC-3′, LAMP1 reverse 5′-AGTGTATGTCCTCTTCC AAAAGC-3′.

### 4.6. Statistical Analysis

The results of Western blotting and MTT were evaluated with a two-tailed Student’s *t*-test using Excel software (Microsoft, Redmond, WA, USA). A *p*-value of 0.05 was considered significant.

## Data Availability

Not applicable.

## Supplementary Materials

The following are available online at https://www.mdpi.com/1422-0067/22/6/2947/s1.
