# Supplementary material for: 6-Azauridine Induces Autophagy-Mediated Cell Death via a p53- and AMPK-Dependent Pathway
Source: Int J Mol Sci. 2021 Mar 14;22(6):2947. doi: 10.3390/ijms22062947 (PMC8000275; doi:10.3390/ijms22062947)
Supplement: Supplementary file 1 [file ijms-22-02947-s001.pdf]

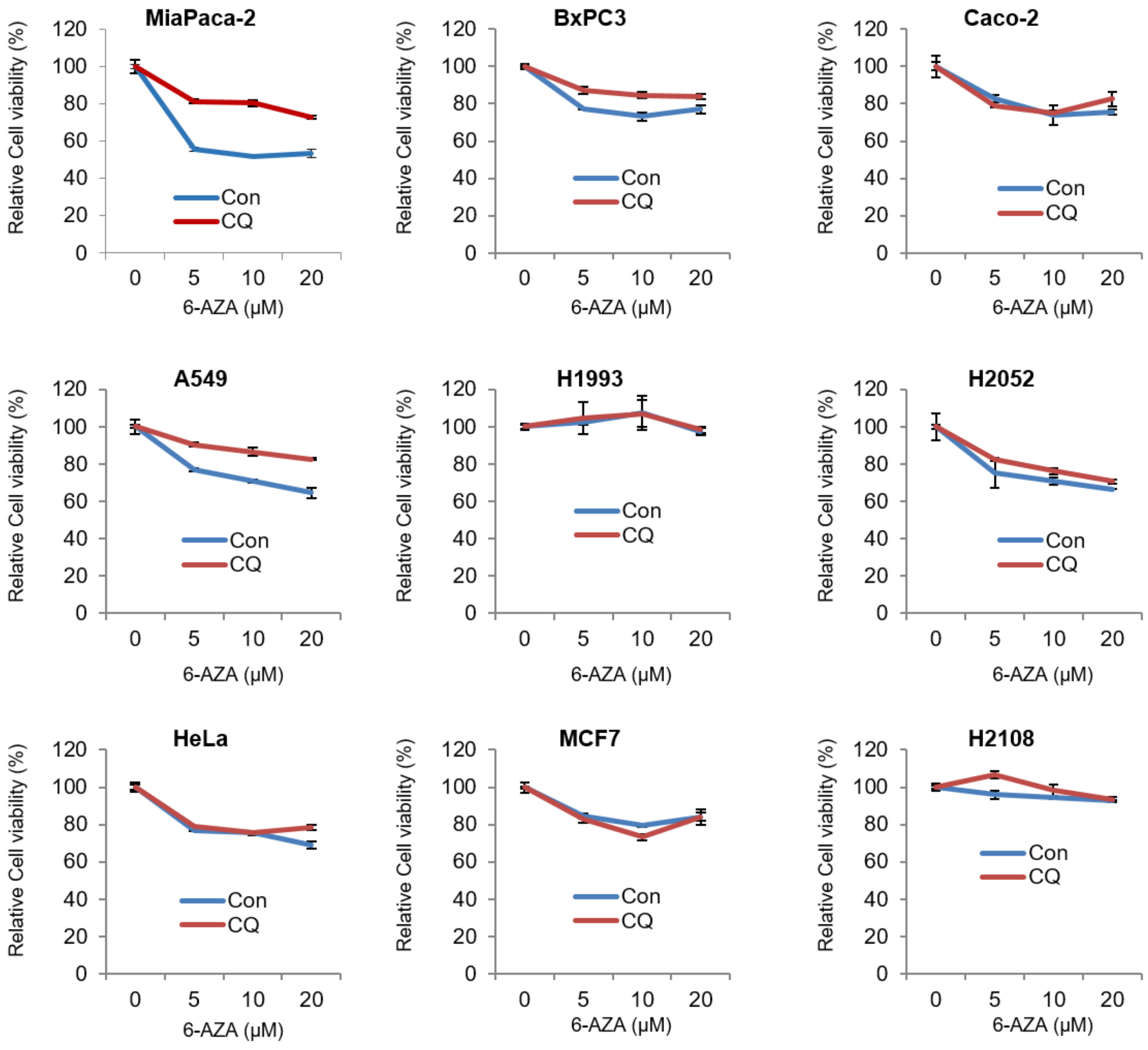

Figure S1. Various cancer cells were treated with the indicated concentration of 6-AZA in the presence or absence of CQ (25 μM). Cell viability was measured using the MTT assay and relative cell viability calculated. Control (Con), and chloroquine treatment (CQ).

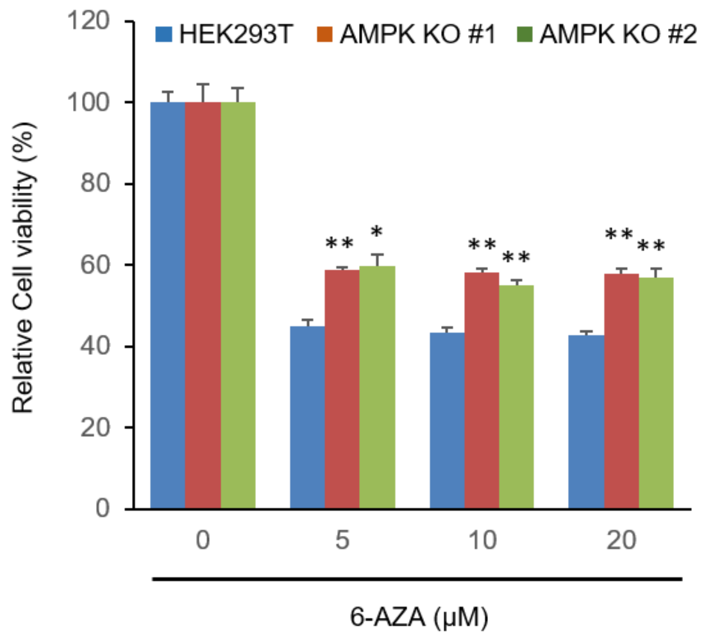

Figure S2. Control and AMPK KO HEK293T cells were treated with the indicated concentration of 6-AZA. Relative cell viability was subsequently measured by the MTT assay. HEK293T vs. AMPK KO cells, * p <0.05, ** p <0.005.

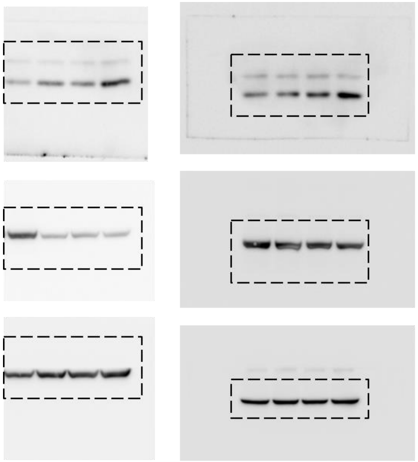

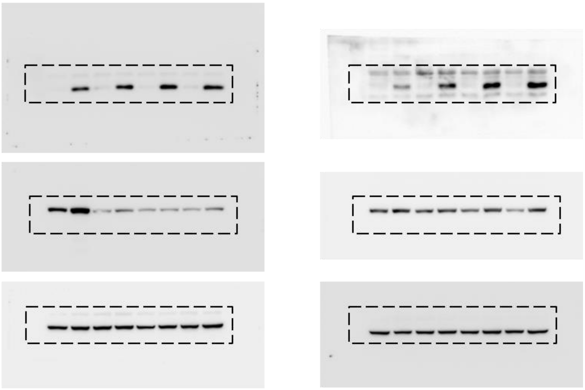

Figure 1

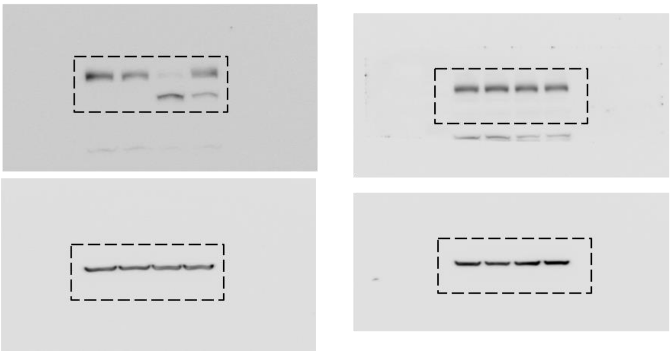

Figure 3

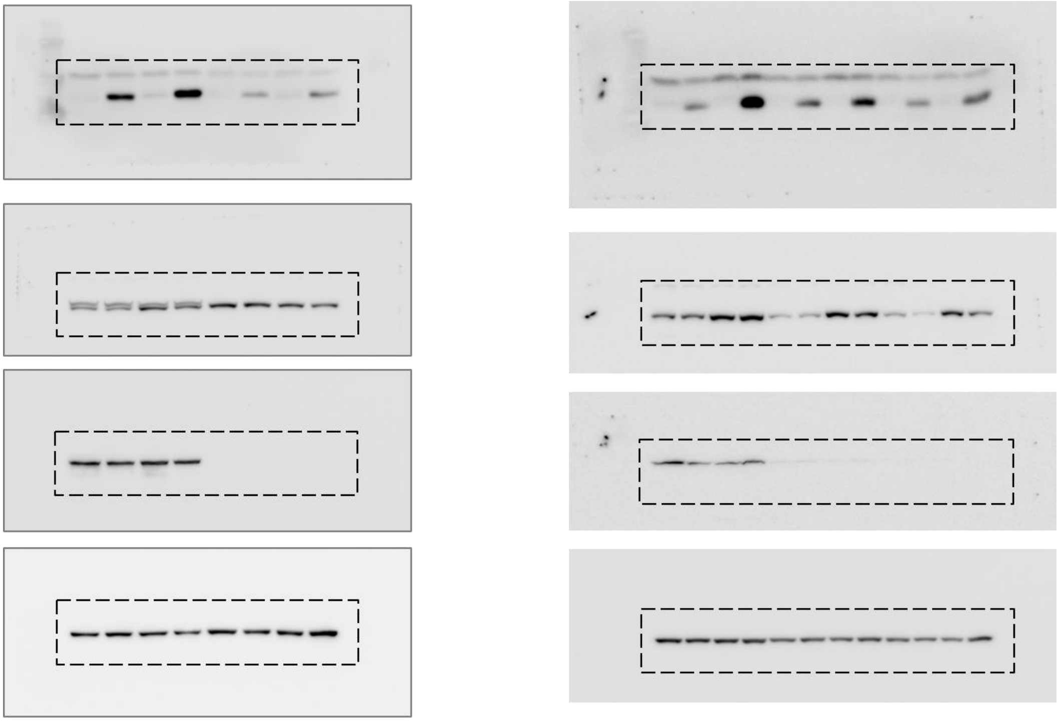

Figure 5

Figure S3. Full length blots cropped for representative figures.
